# Evaluating *Otto the Auto*: Does Engagement in an Interactive Website Improve Young Children’s Transportation Safety?

**DOI:** 10.3390/ijerph14070804

**Published:** 2017-07-19

**Authors:** David C. Schwebel, Anna Johnston, Jiabin Shen, Peng Li

**Affiliations:** 1 Department of Psychology, University of Alabama at Birmingham, 1300 University Blvd, CH 415, Birmingham, AL 35294, USA; ajohn@uab.edu (A.J.); jiabin.shen@nationwidechildrens.org (J.S.); 2 Research Institute at Nationwide Children’s Hospital, 700 Children’s Drive, Columbus, OH 43205, USA; 3 Department of Biostatistics, University of Alabama at Birmingham, 1665 University Blvd, Birmingham, AL 35294, USA; pli@uab.edu

**Keywords:** transportation safety, children, traffic safety, injury prevention, mHealth, eHealth, website, *Otto the Auto*

## Abstract

Transportation-related injuries are a leading cause of pediatric death, and effective interventions are limited. *Otto the Auto* is a website offering engaging, interactive activities. We evaluated *Otto* among a sample of sixty-nine 4- and 5-year-old children, who participated in a randomized parallel group design study. Following baseline evaluation, children engaged with either *Otto* or a control website for 2 weeks and then were re-evaluated. Children who used *Otto* failed to show increases in transportation safety knowledge or behavior compared to the control group, although there was a dosage effect whereby children who engaged in the website more with parents gained safer behavior patterns. We conclude *Otto* may have some efficacy when engaged by children with their parents, but continued efforts to develop and refine engaging, effective, theory-driven strategies to teach children transportation safety, including via internet, should be pursued.

## 1. Introduction

In 2015, transportation-related incidents killed 173 American children ages 4 and 5. Another 50,000 children ages 4 and 5 were injured seriously enough that they required a visit to an emergency department. Together, those injuries inflicted an estimated societal financial burden of just under $1 billion dollars [1]. Transportation-related injuries are the leading cause of death among young American children by a large margin, and remain so through child, adolescent and early adult development.

Public health experts identify multiple ways of reducing transportation-related injuries to children [2,3,4]. Effective means include efforts to develop and implement better road, traffic, vehicle and child restraint system engineering. Efforts also include behavioral strategies to reduce dangerous, distracted and risky driving, and to improve adult supervision of children on and near roadways. Still other efforts, and the current focus, involve attempts to alter the behavior of children themselves.

At a young age, children have less developed cognitive skills, and therefore struggle to accurately judge vehicle speeds and distances, identify safe opportunities to enter roadways, and consistently remember to use appropriate safety equipment and gear when engaging in or near traffic [5]. Without the ability for complex cognitive processing, an alternative strategy to improve young children’s safety is to teach them basic rules they must follow (e.g., cross streets only with an adult; always wear a bicycle helmet). Young children have the cognitive capacity to learn and remember basic safety rules [6], and empirical evidence suggests such rules promote safety and reduce young children’s injury risk to some extent by changing their behavior, although they are not fully sufficient to prevent all injuries [6,7,8,9]. Therefore, even when cognitive functioning is immature, children can and should play a role in keeping themselves safer by learning, remembering and obeying safety rules. The present study used this strategy: we sought to teach children basic rules about transportation safety with the notion that it might reduce injury risk among a sample of 4- and 5-year-old children.

Over the last two decades, pediatric health education has been transformed by the use of technology [10]. Lessons that were previously delivered primarily by parents and teachers can now be disseminated broadly and consistently through computer software and internet websites. An innovator in using this platform for pediatric transportation safety training was the AAA Auto Club, which developed the *Otto the Auto* website in the late 1990s. Interactive computer-based health education has been demonstrated to effectively teach children skills in a range of domains, including asthma [11] and diabetes [12] management, but no published empirical studies evaluate the efficacy of the *Otto the Auto* website. The present study was designed to accomplish that goal. We hypothesized that *Otto* would be an effective tool for teaching children knowledge about transportation safety and to improve their simulated behavior (that is, behavior replicating real-world situations but ethical to measure in a laboratory setting) in transportation settings. We also hypothesized that there would be greater positive effects among children who used the website more often.

## 2. Methods 

### 2.1. Participants

**Figure 1:**
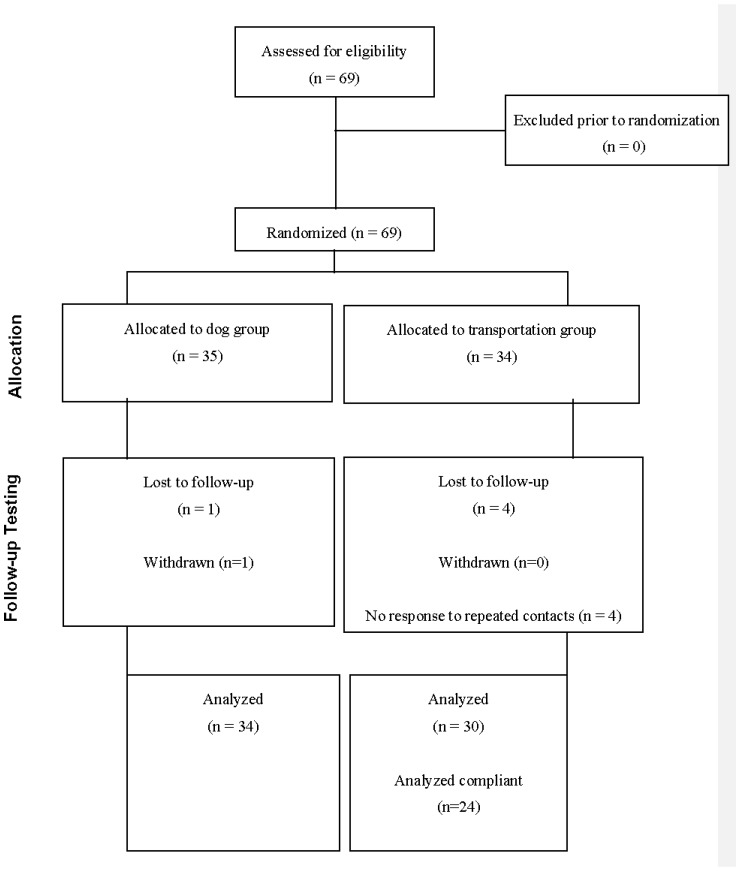
CONSORT flowchart of study enrollment.

Sixty-nine 4- and 5-year-old children were recruited from schools and other community sources in the Birmingham, Alabama area for a parallel group design study. Recruitment occurred between March 2015 and January 2016. Families were required to have a desktop or laptop computer and internet access available at their home for use during the study. No other exclusion criteria beyond child age were applied. Thirty-five were randomly assigned to interact with the *Otto the Auto* website at home and thirty-four assigned to interact with a control website on safety with dogs (see Figure 1 for CONSORT (CONsolidated Standards Of Reporting Trials) flowchart of participation and Table 1 for descriptive data about the sample). Random assignment was conducted by children choosing a group assignment written on folded paper following the baseline assessment. The sample was 48% male and had an average age of 5.06 years (SD = 0.59). It was racially diverse, approximating the racial diversity of the state and region, with 67% of parents identifying their children as White, 25% as African American, and 9% as other races/ethnicities, or as bi-racial or multi-racial. All participants’ parents provided written informed consent, and children provided verbal assent. Families were compensated for their time participating in the study. The study was conducted in accordance with the Declaration of Helsinki, and the protocol was approved by the Institutional Review Board (Ethics Committee) at the University of Alabama at Birmingham (Protocol Number X130802002).

### 2.2. Study Protocol

Families participated in two laboratory visits, one at baseline and a second after an average of about 16 days using either the target transportation safety *Otto the Auto* website or a control website on dog safety at home. Baseline and post-intervention visits comprised detailed evaluations of children’s knowledge and intended behavior in transportation circumstances. Details about both websites and the assessments appear below.

### 2.3. The Transportation Safety Website: Otto the Auto

*Otto the Auto* is an interactive website designed for young children. Introduced in the late 1990s, it engages children with bright colors, catchy music, and various lessons on transportation safety. Children learn about safety while walking, bicycling, and riding in vehicles. Basic rules are taught and children are instructed to wear appropriate safety equipment (e.g., helmets while cycling) and attire (e.g., bright colors while walking). The website includes stories, songs, printable activity sheets, and interactive games. The website was available for public use without charge for at least 10 years, including during active data collection for this study. As of June 2017, it is not available for public use.

### 2.4. The Control Website: Dog Safety

Children randomly assigned to the parallel active control group were exposed to a self-developed website on safety with dogs. We selected this website as a control group because it offered many similarities to our transportation safety website of interest. Like *Otto the Auto*, the dog safety website included interactive games and videos. It taught basic rules about safety with pet dogs and was developmentally-appropriate, engaging, and interactive. It included approximately the same number of games/activities, and targeted a similar stage of child development to teach a similar topic of safety and injury prevention. A detailed analysis of the dog safety website is available elsewhere [13].

### 2.5. Web Use Diaries

Families completed a brief diary entry each time children used the website; diaries were identical for both groups. On the diary, parents reported the amount of time children used the website and whether the child was alone or with a parent while using the website. The parent also reported how much the child enjoyed using the website on a 5-point scale (1 = none to 5 = a lot).

### 2.6. Pre- and Post-Intervention Assessment

Children completed laboratory assessments an average of 15.97 days (SD = 4.71) apart. Between the two assessments, families were instructed to use the randomly-assigned website at home. Laboratory pre- and post-intervention assessments were very similar; details about the tasks used in this report are provided below.

*Demographics*. Parents self-reported family demographic information on a brief questionnaire.

*Traffic Safety Knowledge*. Children completed a 10-item “knowledge” scale orally with a research assistant. Each item was answered dichotomously (yes/no); sample questions include, “Should you cross the street when you see a yellow traffic light?”, “Should you wear a seat belt if you’re only going on a short car ride?”, and “Is it OK to bike right through a road with a stop sign if there are no cars nearby?”. All content was culled from lessons at the *Otto* website. The total number of correct items was summed to create children’s scores.

*Traffic Safety Behavior*. Children engaged in a structured task using dolls and toy vehicles on a small floor-based model village. Modeled after previous work [14,15], the task was designed to simulate and therefore assess children’s behavioral intentions in a variety of situations that replicate the lessons they might have learned on the *Otto the Auto* website. Specifically, children engaged in an interactive “story” guided by researchers. Children were first instructed to choose a doll and to then dress the doll in the “best” outfit and hat/helmet to wear for a bike ride. They then “rode” the character on the bike to the model playground. Following a “play” time at the playground, the child was instructed to safely bicycle back home, eat lunch, and then to choose the safest way to walk to the model library to check out a book. At the end of the simulation, the child was picked up at the library by car and driven to the bakery café for dinner.

Seventeen objective categorical decisions and behaviors were coded live by trained researchers throughout the simulation. As examples, we coded whether the child chose a bright- or dull-colored outfit for the character to bicycle in and whether the doll was given a bicycle helmet to wear or some other alternative (e.g., ball cap). During bicycle trips, we coded whether the character stopped at the stop sign, “looked” for other traffic before crossing the street, and whether the bicycle stayed on the right edge of the road. Similarly, during the pedestrian walk we coded whether the character stayed in the crosswalk and “looked” both ways for traffic. During the car trip, we coded whether the booster seat in the model car was used and whether the seat belt was attached.

### 2.7. Analysis Plan

Data analysis proceeded in three steps. First, we considered self-reported website usage. In both groups, we examined the total and number of times children used the website and their report of how much the children enjoyed the website during their first use. We examined use of the website first because children who did not use the transportation safety website were classified as non-compliant to the intervention and dropped from analysis. Children who did not use the dog safety website were retained in the sample, as non-compliance with the control website, though comparable to non-compliance with the transportation safety website, was not deemed relevant to test the present hypotheses. Second, we tested our primary hypothesis using analysis of covariance (ANCOVA) models on the two primary outcomes, performance on the knowledge scale and behavior in the simulation, controlling for the baseline pre-measures. Third, we conducted general linear regression models evaluating dose response in the intervention group. Specifically, we tested whether greater numbers of exposure to the *Otto the Auto* website were associated with greater improvement on outcome measures among children in the intervention condition.

## 3. Results

Table 1 presents demographic information about the randomized samples. As shown, there were modest differences between the groups on several characteristics, although the only statistically significant demographic difference that emerged across groups was for gender. The transportation safety group had more boys than the dog safety group. Subsequent analyses were run both controlling for gender and without control. Results were very similar so results without controlling for gender are shown.

Table 2 shows self-reported use of the website, divided into use by the child alone, use by the child and parent together, and overall use, plus self-reported enjoyment of the website. Children reported high enjoyment of both the transportation and dog safety websites. Children used the two websites an equivalent amount of times. Six children assigned to the transportation safety group reported no use of the website at all, either alone or with a parent. Those children were considered non-compliant with the intervention and dropped from further analysis. The 24 compliant children reported a minimum of 44 min of use (M = 128.3 min; SD = 84.9; range = 44.0 to 409.5).

Table 3 shows baseline pre-intervention and post-intervention scores for the two outcome measures across the intervention and control groups. As shown, the intervention group experienced a modest increase in scores on both outcome measures. The control group experienced a small increase in knowledge but a small decrease in simulated behavior on outcomes for which they were not exposed to information. Table 4 shows results of inferential ANCOVA analyses examining these changes, with baseline measures controlled. As shown, there was no significant increase in knowledge or simulated behavior among the intervention group compared to children in the control group.

In our final analysis, we considered dose response: did greater exposure to the *Otto the Auto* website yield higher scores on outcome measures? This analysis was conducted only among compliant children in the intervention condition. Four regressions were conducted, one each for total child exposures and times children and parents engaged in the website together for each outcome measure. Child age and baseline scores were entered in the models as covariates. Just one of the regressions yielded a significant effect of website exposure, the model predicting simulated behavior based on times children and parents used the website together (Table 5).

## 4. Discussion

The *Otto the Auto* website was publicly accessible online for over a decade, but no published work documents its efficacy. Our results suggest the website alone is not successful at transmitting knowledge or altering children’s simulated behavior. However, we did discover a significant effect of behavior change when examining the dose effect. Specifically, children who engaged on the *Otto* website more often together with parents achieved greater improvement in their simulated behavior scores than children who less frequently engaged on the website with parents. There are several possible explanations for this finding, but we believe it most likely that parents may have extended the website lessons through their interaction with children while engaged on the website, reinforced the lessons in daily life, and engaged children’s cognitive processing to learn material more richly than those children who learned from the website on their own [16]. These hypotheses are supported by educational research on the role of parents in children’s learning [17,18]. Future research should consider further the process of young children learning safety lessons through internet-based activities, and how parents may reinforce or support learning through joint engagement on websites.

The cognitive task of negotiating traffic environments is complex and demanding for young children [19]. It requires consideration of multiple stimuli in a dynamic situation and may only be accomplished with substantial practice, feedback, and support among children older than our target age group in this study [20]. For young children, instruction of basic rules about how to engage in safety—and reinforcement of those rules along with careful supervision by engaged parents—may be the best way to reduce transportation safety injury risk. Websites such as *Otto the Auto* may facilitate such parenting behavior. Also critical for any public health interventions is incorporation of a theoretical basis that will promote behavior change [21]. A range of theories exist to support behavior change that may reduce child injury risk [22], and such theories must be applied within the context of the population target for change (e.g., young children, older children, parents). On the surface, it is unclear whether *Otto* was developed with a particular health behavior change theory in mind. If there was a lack of a theoretical basis, it may have contributed to poor efficacy of the website in the absence of parental involvement and support.

This study suffered from limitations. One significant weakness was that we studied a website that had been publicly available online for many years without published evaluation data, but it is no longer publicly available (as of July 2017), and officials we communicated with at the AAA Auto Club National headquarters were unaware of any plans for re-release or editing of the website. Given its age, the website used older technology and may be considered as less engaging or out of date compared to more recently-created websites, so it may be unavailable in the future despite the fact that our results suggest it provided some valuable educational material. 

The study also had other limitations. Our sample size was relatively small, and was limited to children ages 4–5 in one geographic location; generalization is unknown. Randomization yielded moderately good matching across groups, although the gender distribution was statistically different across randomized groups and non-statistically-significant differences on other demographic traits emerged also. We relied on self-report diaries for website use. Diary data is widely used in related fields [23,24], but families may have been untruthful on those reports due to poor memory, social desirability, or other reasons. We also relied only on laboratory-based measures to assess outcomes. It would have been unethical to place children in actual traffic situations, but field-based assessment may offer richer outcome measures in future research. It is unclear the extent to which our assessments, including our simulation of real-world behavior, are valid measures of how children may actually behave in traffic. Finally, there was some variation in how many days passed between the baseline and post-intervention assessment (SD = 4.71 days); a longer time period between assessments may have provided more time for exposure to the website for a portion of the sample. 

## 5. Conclusions 

*Otto the Auto* is an engaging website designed to teach children transportation safety. Our randomized trial with a sample of 4- and 5-year-old suggests *Otto* is not effective in teaching 4- and 5-year-old children basic knowledge or skills about transportation safety. However, children who repeatedly engaged in the website with parents did see improvements in their simulated behavior. Thus, the website offers at least some potential advantage and the website content might be updated, extended or expanded, with attention to theoretical and developmental underpinnings, so that it plays a role in our efforts to continue to discover and implement ways to reduce children’s risk of transportation-related injuries.

## Figures and Tables

**Table 1:** Descriptive statistics (M (SD) or %) showing demographic characteristics of the randomized groups.

Variables	Transportation (n = 34)	Dog (n = 35)	*p*
Child’s age (years)	5.1 (0.6)	5.0 (0.6)	0.34 ^a^
Mother’s age (years)	35.4 (5.1)	37.0 (5.3)	0.20 ^a^
Father’s age (years)	39.1 (7.0)	39.1 (6.2)	0.99 ^a^
Gender (% male)	61.8%	34.3%	0.02 ^b^
Ethnicity			0.26 ^c^
African American	10 (30.3%)	6 (17.1%)	
Caucasian	19 (57.6%)	27 (77.1%)	
Other	4 (12.1%)	2 (5.7%)	
Mother’s education			0.09 ^c^
High school diploma or less	2 (5.9%)	0 (0.0%)	
Some college/Associate’s degree	10 (29.4%)	4 (11.4%)	
Bachelor’s degree	9 (26.5%)	15 (42.9%)	
Post-Grad/Graduate degree	13 (38.2%)	16 (45.7%)	
Father’s education			0.79 ^c^
High school diploma or less	5 (15.6%)	3 (8.6%)	
Some college/Associate’s degree	5 (15.6%)	7 (20.0%)	
Bachelor’s degree	9 (28.1%)	12 (34.3%)	
Post-Grad/Graduate degree	13 (40.6%)	13 (37.1%)	
Family Income			0.36 ^c^
Below $40,000	8 (25.8%)	3 (8.8%)	
$40,000–$59,000	6 (19.4%)	6 (17.7%)	
$60,000–$79,000	2 (6.5%)	2 (5.9%)	
$80,000–$99,000	5 (16.1%)	5 (14.7%)	
Above $100,000	10 (32.3%)	18 (52.9%)	
Number of adults in home			0.28 ^c^
1	9 (26.5%)	5 (14.7%)	
2	22 (64.7%)	28 (82.4%)	
3	3 (8.8%)	1 (2.9%)	
Number of children in home			0.73 ^b^
1	5 (14.7%)	7 (20.6%)	
2	20 (58.8%)	17 (50.0%)	
3 or more	9 (26.5%)	10 (29.4%)	

^a^ Two sample *t* test; ^b^ Chi-Square test; ^c^ Fisher’s exact test.

**Table 2:** Website use and enjoyment across groups, and independent samples *t*-test results.

	Transportation M (SD; Range) (n = 30)	Dog M (SD; Range) (n = 34)	*t*-Value (*p*)
Rating of enjoyment—first use (1–5 scale)	4.13 (0.95; 2–5)	3.72 (1.03; 2–5)	1.46 (0.15)
Website use—child alone (count)	1.67 (2.01; 0–7)	0.94 (1.32; 0–4)	1.73 (0.09)
Website use—child and parent together (count)	2.47 (2.46; 0–8)	3.56 (2.21; 0–9)	−1.87 (0.06)
Website use—total child exposure (count)	4.13 (3.63; 0–14)	4.50 (2.79; 0–10)	−0.46 (0.65)

**Table 3:** Mean (standard deviation) and 95% confidence interval for outcome variables across groups.

Outcomes	Transportation (Compliant, n = 24)		Dog (n = 35)	
	Pre-IV	Post-IV	Pre-IV	Post-IV
Knowledge	5.79 (1.32) (5.24, 6.35)	6.33 (1.24) (5.81, 6.86)	5.97 (1.38) (5.49, 6.45)	6.50 (1.29) (6.05, 6.95)
Simulated Behavior	6.65 (3.56) (5.14, 8.15)	6.87 (3.06) (5.54, 8.19)	6.80 (2.68) (5.88, 7.72)	6.50 (2.94) (5.48, 7.52)

**Table 4:** Change in outcome scores (Post-Pre), standard deviation, and 95% confidence intervals for participants, and results of ANCOVA Analysis.

Outcomes	Transportation (Compliant, n = 24)	Dog (n = 35)	*p*
Knowledge	0.54 (1.56) (−0.12, 1.20)	0.52 (1.52) (−0.02, 1.06)	0.804
Simulated Behavior	0.28 (3.65) (−1.30, 1.86)	−0.38 (3.20) (−1.50, 0.73)	0.516

Note: Adjusted for pre-intervention baseline measures.

**Table 5:** Regression Model showing dose response of count of times parent and child used website together on children’s simulated behavior (n = 24).

Parameter	Estimate	Standard Error	Wald 95% CI		*p*
Intercept	−5.262	4.291	−13.673	3.148	0.220
Website exposure	0.459	0.186	0.094	0.825	0.014
Child age	1.684	0.862	−0.005	3.373	0.051
Baseline score	−0.694	0.145	−0.977	−0.411	<0.0001
